# Dissecting the roles of Cse1 and Nup2 in classical NLS‐cargo release in vivo

**DOI:** 10.1111/tra.12759

**Published:** 2020-09-15

**Authors:** Allison Lange, Milo B. Fasken, Murray Stewart, Anita H. Corbett

**Affiliations:** ^1^ Department of Biology Emory University Atlanta Georgia USA; ^2^ Cambridge Biomedical Campus MRC Laboratory of Molecular Biology Cambridge UK

**Keywords:** classical nuclear localization signal, Cse1, importin alpha, nuclear protein export, nuclear protein import, Nup2

## Abstract

The importin α/β transport machinery mediates the nuclear import of cargo proteins that bear a classical nuclear localization sequence (cNLS). These cargo proteins are linked to the major nuclear protein import factor, importin‐β, by the importin‐α adapter, after which cargo/carrier complexes enter the nucleus through nuclear pores. In the nucleus, cargo is released by the action of RanGTP and the nuclear pore protein Nup2, after which the importins are recycled to the cytoplasm for further transport cycles. The nuclear export of importin‐α is mediated by Cse1/CAS. Here, we exploit structures of functionally important complexes to identify residues that are critical for these interactions and provide insight into how cycles of protein import and recycling of importin‐α occur in vivo using a *Saccharomyces cerevisiae* model. We examine how these molecular interactions impact protein localization, cargo import, function and complex formation. We show that reversing the charge of key residues in importin‐α (Arg44) or Cse1 (Asp220) results in loss of function of the respective proteins and impairs complex formation both in vitro and in vivo. To extend these results, we show that basic residues in the Nup2 N‐terminus are required for both Nup2 interaction with importin‐α and Nup2 function. These results provide a more comprehensive mechanistic model of how Cse1, RanGTP and Nup2 function in concert to mediate cNLS‐cargo release in the nucleus.

## Abstract 2

Directional transport of cargoes between the nucleus and cytoplasm is mediated by receptors that bind cargo in one compartment and release cargo into a destination compartment. Cargoes that contain a cNLS are recognized by importin‐α in the cytoplasm. Release factors including the importin‐α export receptor, Cse1, and a nuclear pore complex protein, Nup2, ensure efficient cargo delivery into the nucleus. Interactions defined by previous structural studies are required for productive interactions between importin‐α, Cse1, and Nup2 to occur in vivo.

**Abbreviations**
5‐FOA: 5‐fluoroorotic acid
cNLS: classical nuclear localization signal
DIC: differential interference contrast
GFP: green fluorescent protein
NLS: nuclear localization signal
NPC: nuclear pore complex

## 1 INTRODUCTION

Proteins that are destined for the nucleus bear nuclear localization sequences (NLSs) that are recognized and bound by members of the evolutionarily conserved nuclear transport machinery called karyopherins or importins. Cargo proteins that contain a classical NLS (cNLS) are bound to the major nuclear protein import factor, importin‐β, by the importin‐α adapter, after which cargo/carrier complexes enter the nucleus via nuclear pore complexes (NPCs).^1^, ^2^, ^3^, ^4^ Once in the nuclear interior, cargos are released and the importins are recycled back to the cytoplasm for additional rounds of transport.^5^ The nuclear export of importin‐α is mediated by Cse1/CAS, a member of the exportin class of karyopherins that escorts cargoes from the nucleus to the cytoplasm^6^, ^7^, ^8^, ^9^ and which also is involved in a spectrum of other cellular functions, including chromosome segregation, DNA repair, genome stability and apoptosis.^10^, ^11^, ^12^, ^13^ Together, these transport factors allow for ongoing cycles of nuclear protein import.

In nuclear transport pathways, cargo release is coordinated by the small GTPase, Ran (reviewed in References 14, 15), that is found predominantly in a GDP‐bound state in the cytoplasm and in a GTP‐bound state in the nucleus.^16^, ^17^ This asymmetric distribution results from the compartmentalization of the Ran GTPase activating protein (RanGAP) in the cytoplasm and the Ran guanine nucleotide exchange factor (RanGEF) in the nucleus.^18^, ^19^, ^20^, ^21^, ^22^ As a consequence of this compartmentalization, Ran functions like a switch, controlling whether the transport receptors bind or release their cargo, so that import complexes form in the cytoplasm in the absence of RanGTP and are dissociated in the nucleus in the presence of RanGTP. Conversely, export complexes form in the presence of RanGTP and dissociate upon hydrolysis of RanGTP to RanGDP in the cytoplasm.^1^, ^23^ This GTP‐dependent switch imparts directionality on the transport pathway.

The classical nuclear import pathway is the most comprehensively studied system of nuclear protein import and is mediated by a heterodimer consisting of importin‐β and an adaptor protein, importin‐α, that binds to cargo proteins containing a cNLS.^2^ Importin‐α is constructed from an N‐terminal importin‐β‐binding (IBB) domain, a central ARM repeat domain, and a short C‐terminal domain^24^ and recognizes cNLS motifs that consist of either one (monopartite) or two (bipartite) stretches of basic amino acids.^2^ Monopartite cNLSs bind to a major NLS‐binding pocket on the inner surface of importin‐α and bipartite cNLSs bind to both the major pocket and a minor NLS‐binding pocket.^24^ Classical protein import cargoes bind to the heterodimeric importin‐α/β import receptor that translocates through NPCs into the nucleus, where the complex is dissociated by RanGTP binding to importin‐β, which triggers a conformational change^25^, ^26^, ^27^ that releases the importin‐β‐binding (IBB) domain and generates a transient cNLS‐cargo/importin‐α complex. The freed IBB domain, which is autoinhibitory and contains a cNLS‐like sequence,^28^, ^29^ then competes for binding with the cNLS, releasing the protein cargo into the nucleus.^24^, ^30^, ^31^

The effect of RanGTP coupled with the autoinhibitory domain is sufficient to modulate the affinity of cNLS‐cargo for importin‐α in vitro; however, the spontaneous dissociation of the cNLS‐cargo/importin‐α/importin‐β complex occurs too slowly to account for the rapid release of protein cargo required in vivo.^25^ Two other karyopherin release factors (KaRFs) have been implicated in the release of cNLS‐cargo: the export factor for importin‐α, Cse1, and the nucleoporin, Nup2.^25^, ^32^ Cse1, a member of the β‐karyopherin superfamily of receptors,^33^, ^34^ is a helicoidal molecule constructed from 20 tandem HEAT repeats and has an N‐terminal RanGTP‐binding domain.^34^ Nup2 is a NPC protein (nucleoporin) constructed from an N‐terminal domain, a central natively‐unfolded domain that contains phenylalanine‐glycine (FG) repeats and which bind both importin‐β and the nucleoporin Nup60, and a C‐terminal Ran‐binding motif.^35^, ^36^, ^37^, ^38^ Cse1 and Nup2 can both accelerate cNLS‐cargo release in vitro^25^ and mutations in both *CSE1* and *NUP2* cause synthetic growth defects when paired with mutations in the importin‐α gene, *SRP1*, that alter autoinhibitory function, pointing to their involvement in nuclear protein delivery in vivo.^28^ Studies in mammalian cells that support this model employed single‐molecule fluorescence resonance energy transfer and particle tracking in permeabilized HeLa cells. These studies show that the complex between importin‐α and CAS, the human ortholog of Cse1,^12^ forms in the nuclear basket region of NPCs and appears to also involve NUP50, the metazoan homolog of Nup2,^39^ which has been proposed to orchestrate cargo dissociation,^39^, ^40^ although the choreography of the individual proteins and complexes is quite complicated.^32^

**FIGURE 1:**
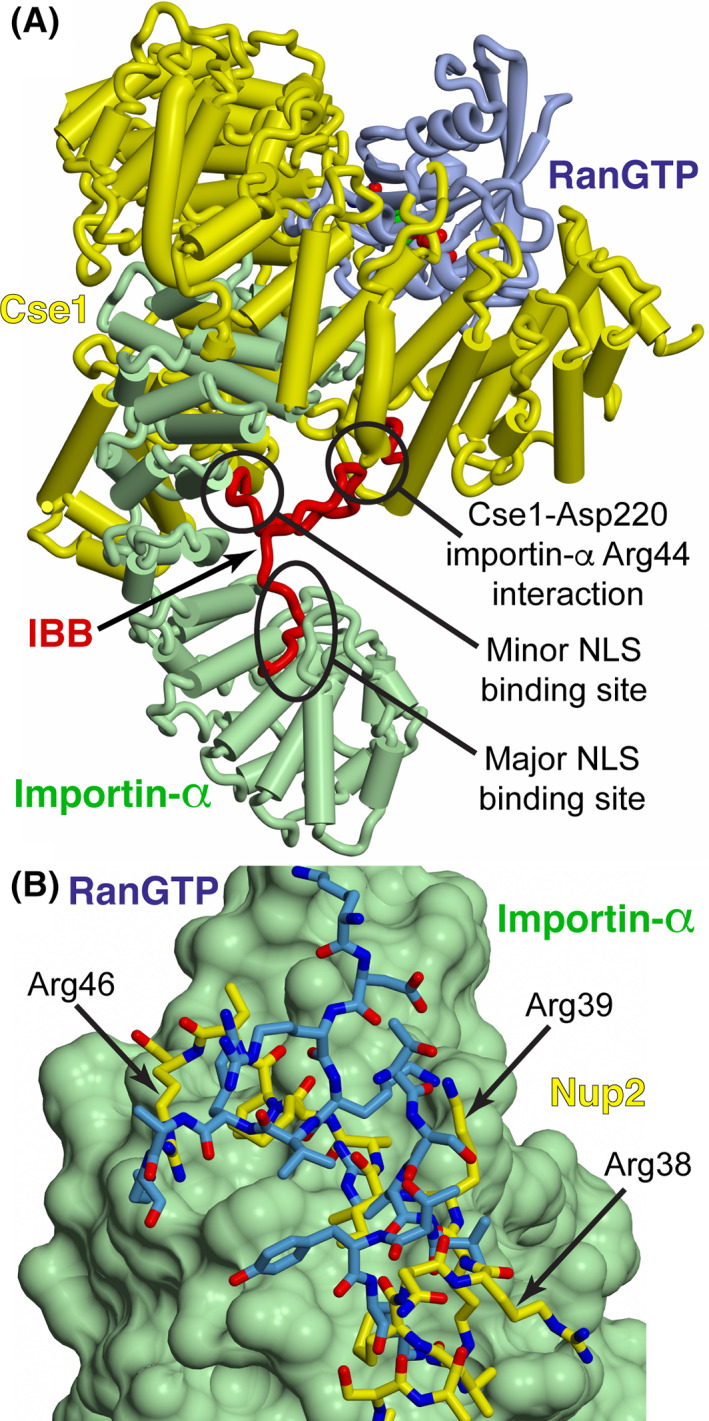
A, Schematic of the importin‐α/Cse1/RanGTP complex.^5^ Importin‐α is shown in green with the IBB domain shown in red, Cse1 is shown in yellow, and RanGTP is shown in blue (adapted from PDB 1WA5^5^). When importin‐α is bound by Cse1‐RanGTP, the autoinhibitory IBB domain is inserted into the major and minor NLS‐binding sites on importin‐α. The interaction between Cse1 Asp220 and importin‐α Arg44 is central to the generation of the complex.^5^ In the *cse1‐1* mutant, in which importin‐α export is impaired, Asp220 is changed to Asn. B, RanGTP (blue) in the importin‐α/Cse1/RanGTP complex and the Nup2 N‐terminus (yellow) overlap when bound to a region at the importin‐α C‐terminal region (shown as a green surface) and so cannot bind simultaneously. The structures shown are based on PDB 2C1T (Nup2:importin‐α complex^41^) and PDB 1WA5 (importin‐α/Cse1/RanGTP complex^5^)

Crystal structures of the Cse1/RanGTP/importin‐α complex and of the Nup2/importin‐α complex^5^, ^40^, ^41^ provide a detailed basis for how Cse1 and Nup2 influence cargo release in the nucleus. The crystal structure of importin‐α bound to its export receptor Cse1/RanGTP^5^ shows that RanGTP and the C‐terminus of importin‐α are nestled between two arched regions of Cse1 (Figure 1A). The IBB domain of importin‐α then folds back over the body of the molecule and binds to the major and minor cNLS‐binding pockets. Importantly, the IBB domain also interacts with Cse1 in two key regions. First, the extreme N‐terminus of the autoinhibitory IBB domain interacts with Cse1, holding the entire molecule in a “closed” position, and second, a region between the two basic clusters of the IBB domain interacts with the N‐terminal Cse1 arch. These studies indicate that the interactions of the IBB domain with Cse1 are critical to efficient cNLS‐cargo delivery into the nucleus and to recycling of importin‐α back to the cytoplasm. These interactions could function in two important ways: first, by facilitating the release of cNLS‐cargo by clamping importin‐α in a closed state, thereby reinforcing the autoinhibitory function of the IBB domain and preventing cargo from rebinding; and, second, by providing a mechanism for Cse1 to distinguish between cargo‐bound and cargo‐free importin‐α, thereby preventing futile transport rounds of transport resulting from the export of importin‐α still bound to cargo.

The crystal structure of the N‐terminus of Nup2 bound to ΔIBB importin‐α^40^, ^41^ shows that Nup2 binds to importin‐α in two main regions: one near the center of the molecule that partially overlaps the minor cNLS‐binding pocket, and one on the importin‐α C‐terminus that overlaps the Cse1/RanGTP‐binding site (Figure 1B). Amino acid substitutions in the mammalian homolog of Nup2, NUP50/NPAP60, combined with in vitro binding studies suggested that basic residues in both binding regions of Nup2 are involved in binding importin‐α and dissociating cNLS‐cargo.^40^ These results indicate that the function of Nup2 is analogous to the autoinhibitory importin‐α IBB domain, binding to the C‐terminus of importin‐α, then folding over to compete with cNLS‐cargo for binding, thereby contributing to cargo release.

In this study, we test the Cse1/RanGTP/importin‐α and Nup2/importin‐α structural models in vivo by changing residues essential for complex formation and assaying their effects on protein localization, cargo import, function and complex formation. We find that reversing the charge of Arg44 in importin‐α or Asp220 in Cse1, residues that crystal structures indicate are critical for interaction between the IBB domain of importin‐α and the N‐terminal arch of Cse1,^5^ results in loss of function of the respective proteins. Furthermore, these reversal‐of‐charge variants in either importin‐α or Cse1 impair complex formation both in vitro and in vivo. Substitutions of basic residues that target two regions of the Nup2 N‐terminus that are predicted to interact either with the minor cNLS‐binding pocket or with the C‐terminus of importin‐α, disrupt both the function of Nup2 and its interaction with importin‐α. These results are consistent with the structures of Cse1/RanGTP/importin‐α and Nup2/importin‐α being representative of the state of the complexes in vivo, providing insight into a mechanistic model explaining how these proteins function in concert to ensure directional transport events.

## 2 RESULTS

### 2.1 Importin*‐*α Arg44 and Cse1 Asp220 are critical for importin‐α/Cse1/RanGTP complex formation

Both the crystal structure^5^ of importin‐α bound to Cse1/RanGTP (Figure 1A) and previous in vitro binding studies,^5^ indicate that importin‐α Arg44 and Cse1 Asp220 are crucial for formation of the tertiary complex consisting of Cse1/RanGTP/importin‐α. To test this hypothesis in vivo, we created amino acid substitutions in both importin‐α and Cse1 that either neutralize or reverse the charge of the relevant amino acid (R44A, R44E, R44D or R44K in importin‐α and D220A, D220R, D220K or D220N in Cse1) and assessed the function, localization and interactions of these variant proteins.

**FIGURE 2:**
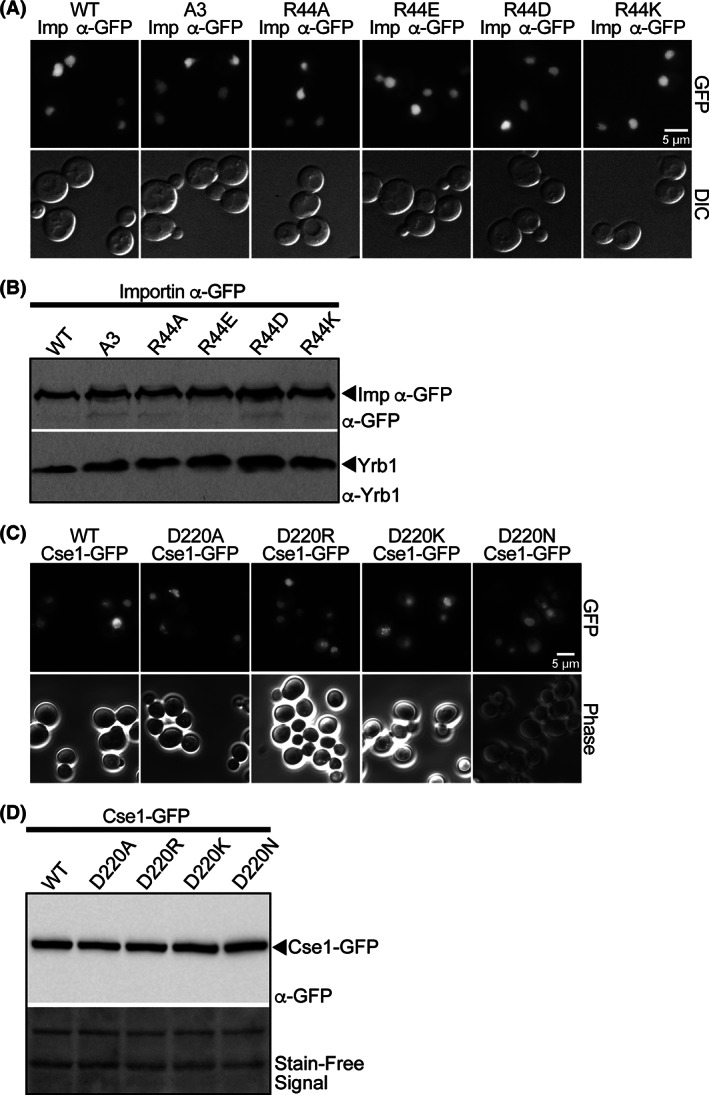
A, Wild‐type cells expressing importin‐α‐GFP, A3 (K54A, R55A and R56A) importin‐α‐GFP,^29^ or each of the Arg44 importin‐α‐GFP variants were examined by direct fluorescence microscopy (GFP). Corresponding differential interference contrast (DIC) images are shown. Scale bar is 5 μm. B, The importin‐α‐GFP variants are expressed at approximately equal levels. Lysates from wild‐type cells expressing each of the importin‐α‐GFP variants indicated were probed with anti‐GFP antibodies to detect the fusion proteins and with anti‐Yrb1 antibody^50^ as a loading control. C, Wild‐type cells expressing Cse1‐GFP or each of the Asp220 Cse1‐GFP variants were examined by direct fluorescence microscopy (GFP). Corresponding phase contrast images are shown. Scale bar is 5 μm. D, Cse1‐GFP variants are expressed at approximately equal levels. Lysates from wild‐type cells expressing each of the Cse1‐GFP variants indicated were probed with anti‐GFP antibodies to detect the fusion proteins. The Stain‐Free signal to detect total protein serves as a loading control

The changes introduced into the N‐terminus of importin‐α at amino acid Arg44 lie within the IBB region and could potentially disrupt the interaction with importin‐β, preventing these importin‐α variants from entering the nucleus. As Cse1 interacts with importin‐α after they both reach the nucleus, this potential mislocalization could preclude interaction between importin‐α and Cse1. To verify that the importin‐α amino acid substitutions created do not affect the localization of these variants, each importin‐α variant was tagged at the C‐terminus with GFP and the resulting fusion proteins were visualized in wild‐type yeast cells (Figure 2A). Wild‐type importin‐α‐GFP and all of the importin‐α‐GFP variants are localized to the nucleus at steady‐state, suggesting that the interaction with importin‐β is not affected in any of these importin‐α variants. Immunoblotting shows that all of the importin‐α‐GFP proteins are expressed at approximately equal levels (Figure 2B). Analogous to importin‐α, the amino acid changes introduced into Cse1 at Asp220 could alter the localization or stability of the Cse1 protein. To examine the localization and levels of the Cse1 variants (D220A, D220R, D220K or D220N), we used a Cse1‐GFP fusion protein, which has been previously shown to localize to the nuclear rim^8^ in a manner similar to that observed for endogenous Cse1.^9^ As shown in Figure 2C, each of the Cse1 variants analyzed localizes to the nuclear rim. Furthermore, the level of each Cse1‐GFP variant is comparable to wild‐type Cse1‐GFP (Figure 2D). These experiments demonstrate that the amino acid changes analyzed in both importin‐α and Cse1 do not grossly alter either the steady‐state localization or levels of these key nuclear transport factors, suggesting that these variants can be employed to explore the specific requirement for these residues (Arg44 in importin‐α and Asp220 in Cse1) for the function of these proteins.

**FIGURE 3:**
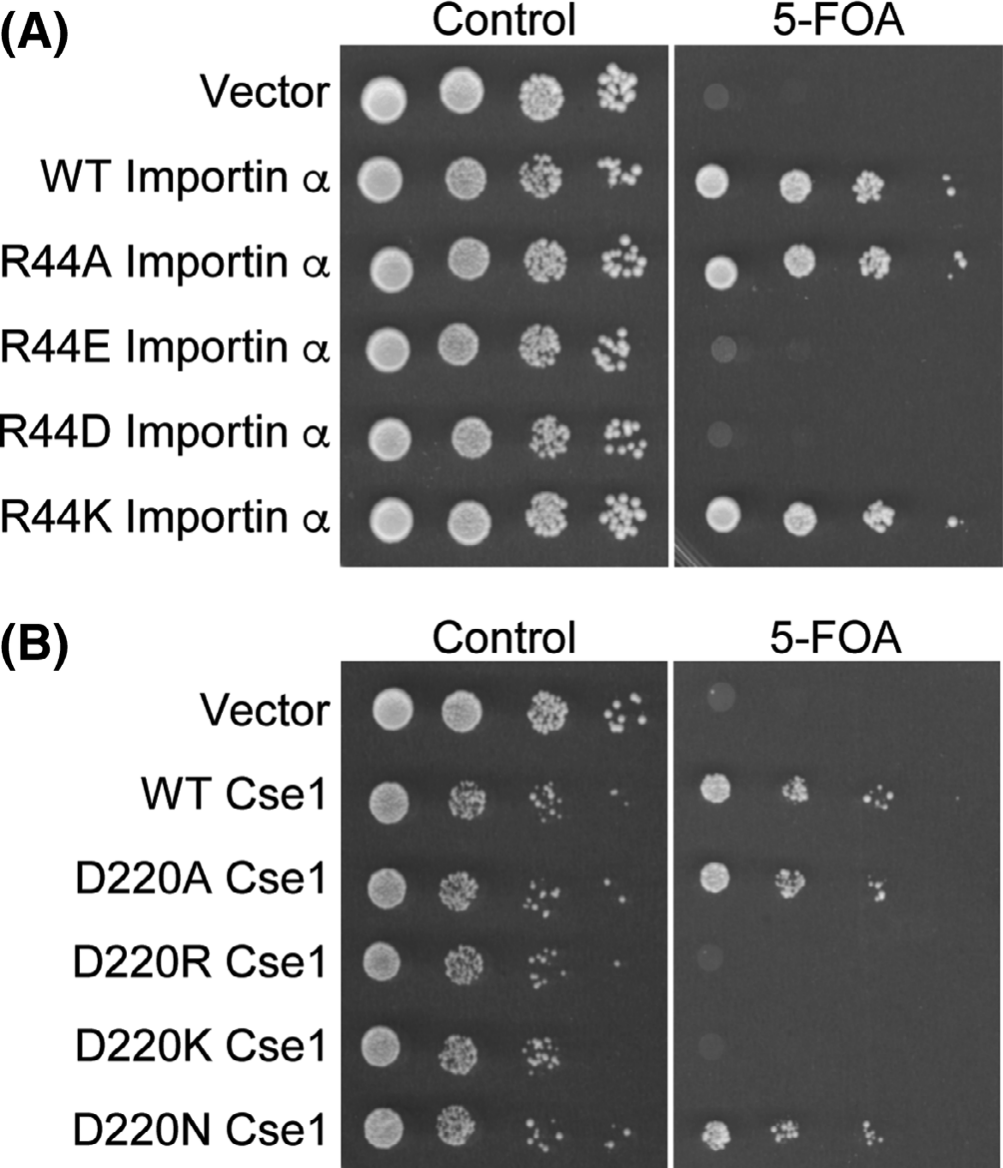
Charged residues that mediate the importin‐α‐Cse1 interaction are critical for the function of each protein in vivo. Protein function in vivo was assessed by a plasmid shuffle assay^42^ as described in Section 4. A, *ΔSRP1* cells maintained by a plasmid encoding wild‐type importin‐α and expressing either wild‐type or importin‐α variants were serially diluted, spotted onto control plates or 5‐FOA plates, which select against the importin‐α maintenance plasmid, and grown at 30°C for 3 days. Importin‐α variants in which the charge of Arg44 is reversed (R44E or R44D) are not able to function as the sole copy of the essential importin‐α protein. B, *ΔCSE1* cells maintained by a plasmid encoding wild‐type Cse1 and expressing either wild‐type or Cse1variants were serially diluted, spotted onto control or 5‐FOA plates, and grown at 30°C for 3 days. Cse1 variants in which the charge of Asp220 is reversed (D220R or D220K) are not functional as the sole copy of the essential Cse1 protein

The function of the importin‐α and Cse1 variants in vivo was assessed using plasmid shuffle assays (Figure 3A,B). Cells lacking the gene for importin‐α, *SRP1*, or the gene for Cse1, *CSE1*, and containing wild‐type *SRP1* or *CSE1 URA3* maintenance plasmids were transformed with plasmids encoding wild‐type importin‐α or Cse1, variant importin‐α or Cse1, or vector alone and were plated on control plates or plates containing 5‐fluoroorotic acid (5‐FOA) to select against the *URA3*‐containing maintenance plasmid.^42^ *SRP1* and *CSE1* are both essential,^13^, ^43^ so only cells with a functional copy of the relevant gene are viable. As shown in Figure 3A, *ΔSRP1* cells expressing R44A or R44K importin‐α grow like wild‐type cells; however, cells expressing the charge reversal variants, R44E or R44D importin‐α, as the sole copy of importin‐α are unable to grow. Similarly, *ΔCSE1* cells expressing D220A or D220N Cse1 grow like wild‐type cells, but cells expressing D220R and D220K Cse1 are inviable (Figure 3B). These results are consistent with reversing the charge of the importin‐α Arg44 or Cse1 Asp220 interrupting an essential interaction between these proteins.

### 2.2 Interaction of importin‐α with Cse1 in vivo

To assay the interaction between importin‐α and Cse1 in vivo, we exploited the fact that Cse1 is the export receptor for importin‐α^8^, ^9^, ^44^ and so employed the steady‐state localization of importin‐α as a measure of the productive in vivo interaction between the two proteins. In cells with wild‐type levels of Cse1, importin‐α‐GFP is localized to the nucleus; however, in cells with elevated levels of Cse1, importin‐α‐GFP is localized throughout the cell, presumably because the increased levels of Cse1 promote more rapid recycling of importin‐α‐GFP to the cytoplasm.^29^ Importantly, this Cse1‐mediated export of importin‐α‐GFP requires a physical interaction between importin‐α and Cse1.^5^

**FIGURE 4:**
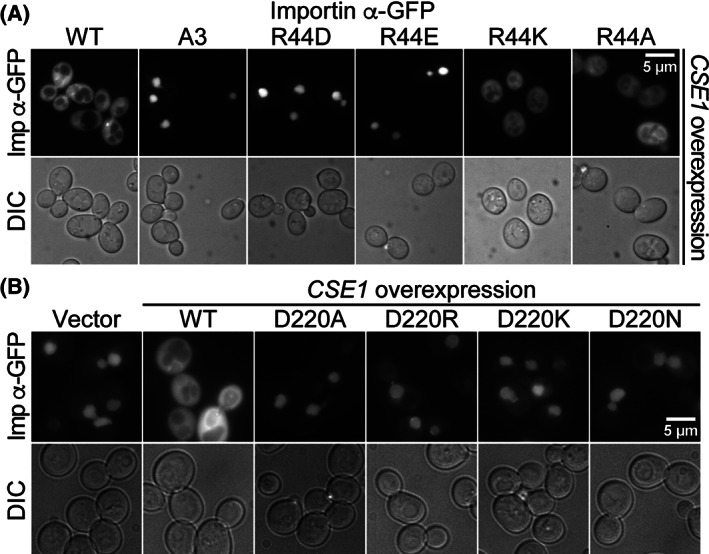
A productive interaction between importin‐α and Cse1 is required for recycling of importin‐α to the cytoplasm. A, Wild‐type cells expressing each of the importin‐α‐GFP variants indicated were transformed with a plasmid to overexpress Cse1. Cells were examined by direct fluorescence microscopy to assess the localization of importin‐α‐GFP. Corresponding differential interference contrast (DIC) images are shown. B, Wild‐type cells expressing wild‐type importin‐α‐GFP were transformed with plasmids that overexpress the indicated Cse1 variants and examined by direct fluorescence microscopy (Imp‐α‐GFP). Corresponding DIC images are shown. Scale bar is 5 μm

Figure 4A shows the localization of the importin‐α‐GFP variants in cells that overexpress wild‐type Cse1. In cells overexpressing Cse1, wild‐type importin‐α‐GFP is localized throughout the cell, whereas a control protein, A3 importin‐α‐GFP, which has reduced binding to Cse1 due to a defect in cargo release,^28^ remains nuclear. The R44A and R44K importin‐α‐GFP variants are also localized throughout the cell, indicating that export of these variants is facilitated by excess Cse1 and consistent with a functional interaction with Cse1 being maintained. In contrast, R44D and R44E importin‐α‐GFP both remain localized primarily to the nucleus, even in cells in which Cse1 is overexpressed, indicating that reversing the charge of the Arg44 residue of importin‐α impairs the interaction with Cse1 in vivo.

The converse experiment was performed to assess the requirement for Cse1 Asp220 in the interaction with importin‐α in vivo. For this experiment, wild‐type importin‐α‐GFP was visualized in cells overexpressing wild‐type or Asp220 Cse1 variants (Figure 4B). As expected, importin‐α‐GFP is localized to the cytoplasm in cells overexpressing wild‐type Cse1. However, in cells overexpressing D220A, D220R, D220K or D220N Cse1, importin‐α‐GFP is restricted to the nucleus, consistent with a requirement for Cse1 Asp220 for productive interaction with wild‐type importin‐α in vivo.

**FIGURE 5:**
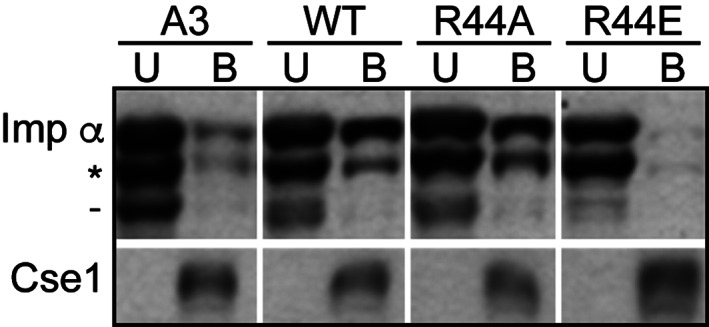
The interaction between importin‐α and Cse1. A, In vitro binding between variants of importin‐α‐myc and Cse1‐GST was examined as described in Section 4. Unbound (U) and Bound (B) fractions were probed with α‐myc antibodies to detect importin‐α‐myc and with α‐GST antibodies to detect Cse1‐GST

To confirm that altering the charge of importin‐α Arg44 impacts the interaction with Cse1, recombinant wild‐type Cse1‐GST was incubated with yeast cell lysate expressing wild‐type or variant importin‐α‐myc (Figure 5). The variants of importin‐α used were A3 importin‐α, a control that has a defect in cNLS‐cargo release, which prevents interaction with Cse1^29^; R44A importin‐α, which neutralizes the charge of the residue; and R44E importin‐α, which reverses the charge of the residue. As expected,^29^ A3 importin‐α exhibits much lower binding to Cse1 than wild‐type importin‐α, whereas R44A importin‐α shows binding to Cse1 comparable to wild‐type importin‐α. However, R44E importin‐α exhibits almost no detectable binding to Cse1 in this assay, indicating that reversing the charge of the Arg44 of importin‐α disrupts the interaction between importin‐α and Cse1 in vitro.

### 2.3 Function of the interaction of the Nup2 N‐terminus with importin‐α

Before importin‐α can be recycled to the cytoplasm, cargo release into the nucleus must occur. The nucleoporin Nup2 has been implicated in this cNLS‐cargo release.^25^, ^41^ Previous studies have suggested that the N‐terminus of Nup2 may provide an initial docking site along the Cse1/RanGTP‐mediated importin‐α export pathway^35^ and have shown that the N‐terminal 50 residues of Nup2 are required for Nup2 function in vivo^41^, both for proper Nup2‐dependent recycling of importin‐α to the cytoplasm,^41^ and for binding of Nup2 to importin‐α in vitro^38^, ^41^. To test whether the basic residues in Nup2 are required for Nup2 function and to test the model for Nup2‐mediated cargo release in vivo, we engineered a series of amino acid substitutions in the N‐terminus of Nup2 to create three Nup2 variants (Nup2 K3E/R4D; Nup2 R38A/R39A/R46A/R47A; and Nup2 K3E/R4D/R38A/R39A/R46A/R47A). For each of these variants, we assessed how the amino acid changes influence the localization and function of Nup2 as well as the productive interaction of Nup2 with importin‐α. The K3E and R4D amino acid substitutions target the first importin‐α binding site within Nup2, which is proposed to bind to the C‐terminus of importin‐α.^41^ The R38A, R39A, R46A and R47A amino acid substitutions target the second binding site within Nup2, which interacts with the minor cNLS‐binding pocket in importin‐α.^41^ R38A, R39A, R46A and R47A target the second binding site within Nup2, which is proposed to interact with C‐terminus of importin‐α^41^ and bind at a site overlapping the RanGTP binding site (see Figure 1B). Basic residues in each of these regions are conserved between *Saccharomyces cerevisiae* Nup2 and NUP50/NPAP60, the mammalian ortholog of Nup2.^40^

### 2.4 Localization of Nup2‐GFP variants

**FIGURE 6:**
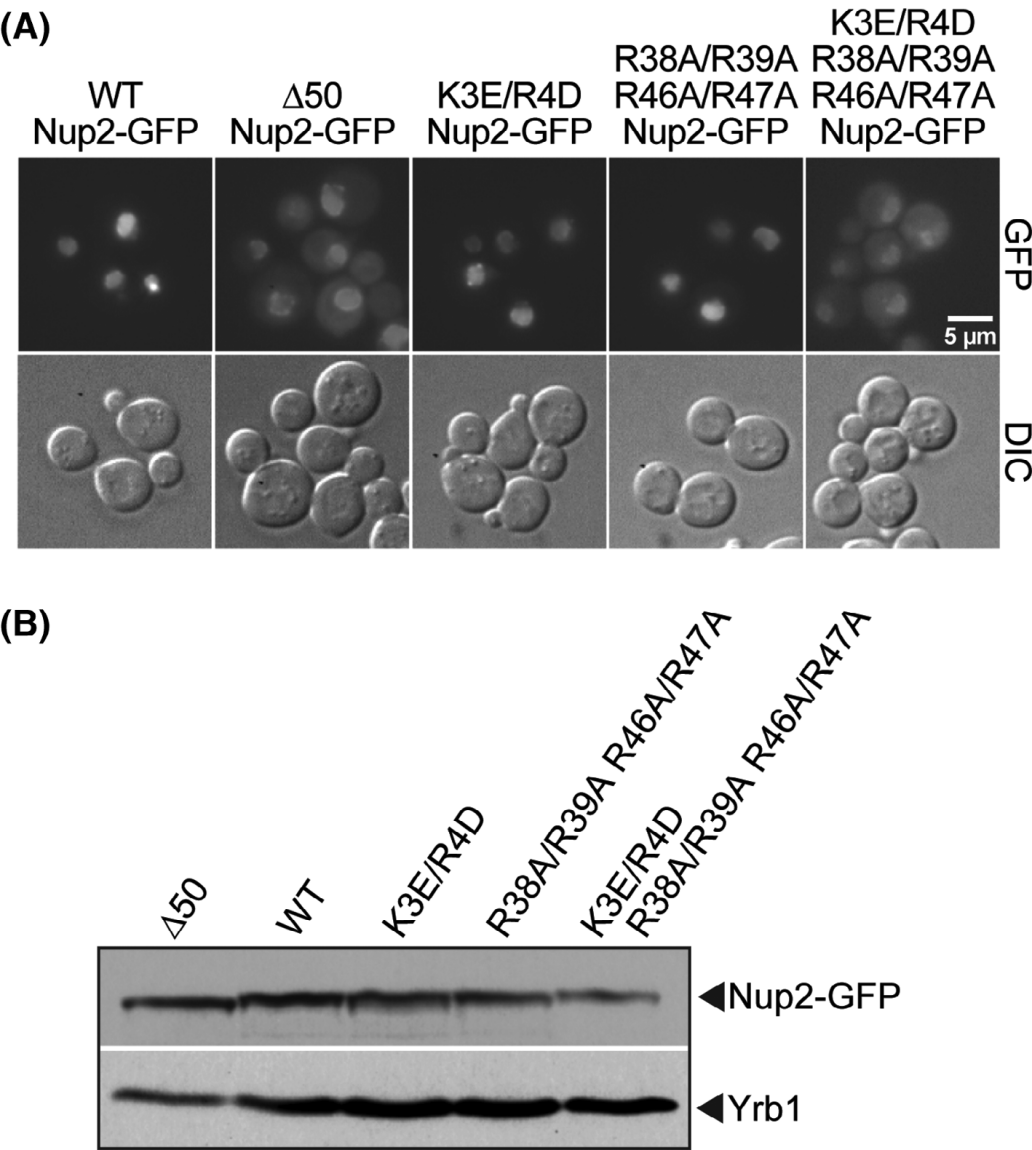
Conserved residues within the N‐terminus of Nup2 hypothesized to be critical for interaction with importin‐α. A, Wild‐type cells expressing wild‐type Nup2‐GFP, Δ50 Nup2‐GFP, or one of the Nup2‐GFP variants were examined by direct fluorescence microscopy (GFP). Corresponding DIC images are shown. Scale bar is 5 μm. B, The Nup2‐GFP variants are expressed at approximately equal levels. Lysates from wild‐type cells expressing each of the Nup2‐GFP variants were probed with anti‐GFP antibodies to detect the Nup2 fusion proteins and with anti‐Yrb1 antibody^50^ as a loading control

Nup2 interacts with importin‐α in the nucleus as a cargo release factor,^25^, ^41^ so it is important to assess whether these variant Nup2 proteins can enter the nucleus. To assess the localization of the Nup2 variants, GFP was fused to the C‐terminus of Nup2 and the resulting fusion proteins were visualized in wild‐type cells (Figure 6A). Wild‐type Nup2‐GFP, K3E/R4D Nup2‐GFP and R38A/R39A/R46A/R47A Nup2‐GFP all localize to the nucleus. Cells expressing Δ50 Nup2‐GFP or K3E/R4D/R38A/R39A/R46A/R47A Nup2‐GFP do show some cytoplasmic signal, but still show significant nuclear localization of the Nup2‐GFP variants. Immunoblotting verifies that the Nup2‐GFP variants are expressed at approximately equal levels (Figure 6B).

### 2.5 In vivo function of Nup2 variants

**FIGURE 7:**
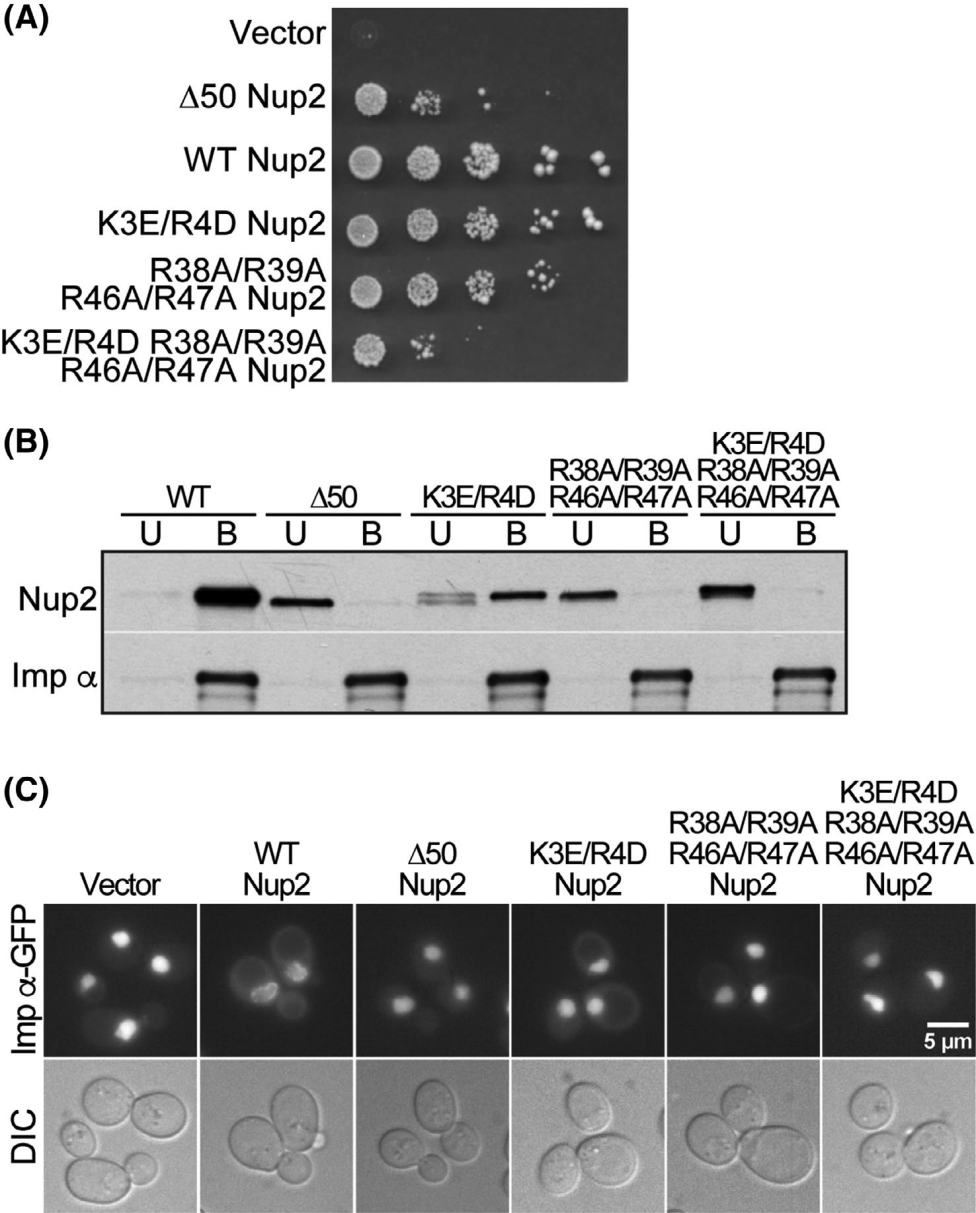
Conserved residues within the N‐terminus of Nup2 are required for proper Nup2 function in vivo and for binding to importin‐α in vitro. A, Nup2 variant protein function in vivo was assessed by a plasmid shuffle assay^42^ as described in Section 4. Δ*NUP2*/Δ*NUP133* cells (ACY1552) maintained by a plasmid encoding wild‐type Nup2 and expressing wild‐type Nup2, Δ50 Nup2, or mutant Nup2 were spotted onto 5‐FOA plates and grown at 25°C. Cells from the 5‐FOA plates were grown to saturation at 25°C, serially diluted, spotted onto selective plates, and grown at 30°C. B, In vitro binding between variants of Nup2‐GFP and importin‐α‐myc was assessed by co‐immunoprecipitation as described in Section 4. The unbound (U) and bound (B) fractions were probed with α‐GFP antibodies to detect the Nup2‐GFP and with α‐myc antibodies to detect the importin‐α‐myc. C, *ΔNUP2* cells transformed with wild‐type Nup2, Δ50 Nup2, or one of the Nup2 variants and expressing integrated importin‐α‐GFP were examined by direct fluorescence microscopy (imp‐α‐GFP). Corresponding DIC images are shown. Scale bar is 5 μm

The function of the Nup2 variants was analyzed using a plasmid shuffle assay^42^ using *ΔNUP2/ΔNUP133* cells containing a wild‐type *NUP2 URA3* maintenance plasmid. This double mutant was employed because *NUP2* is not an essential gene, but the combination of *ΔNUP2* and *ΔNUP133* is synthetically lethal at 30°C^45^, creating a genetic background in which Nup2 function is essential. Cells were transformed with plasmids encoding wild‐type Nup2, Nup2 variants or vector alone, serially diluted and spotted on 5‐FOA plates. Cells from the 25°C 5‐FOA plate were grown to saturation, diluted, spotted on leu‐ glucose plates, and incubated at 30°C (Figure 7A). As observed previously,^41^ control cells expressing Δ50Nup2 grow more slowly than cells expressing wild‐type Nup2. Cells expressing the Nup2 variants show a range of growth phenotypes between those of wild‐type and Δ50 Nup2. The K3E/R4D Nup2 cells show a slight growth defect, R38A/R39A/R46A/R47A Nup2 cells show an intermediate growth phenotype, and the combination K3E/R4D/R38A/R39A/R46A/R47A Nup2 mutant cells show markedly slow growth that is comparable to cells expressing Δ50 Nup2.

### 2.6 Interaction between Nup2 and importin‐α in vitro

The extent to which altering either of the two basic regions within the N‐terminus of Nup2 impairs binding to importin‐α was assessed using an in vitro co‐immunoprecipitation assay. Cells expressing both wild‐type importin‐α‐myc and a variant of Nup2‐GFP were incubated with c‐myc beads after which bound and unbound fractions were assessed by immunoblotting (Figure 7B). As expected, wild‐type Nup2 binds importin‐α, but Δ50 Nup2 shows no binding to importin‐α. K3E/R4D Nup2, R38A/R39A/R46A/R47A Nup2 and K3E/R4D/R38A/R39A/R46A/R47A Nup2 all show decreased binding to importin‐α, with the greatest impairment seen for K3E/R4D/R38A/R39A/R46A/R47A Nup2, where no binding to importin‐α is detected. These results recapitulate the in vivo function data and are consistent with the basic residues in both pockets of Nup2 contributing to Nup2 binding to importin‐α in vitro, although the basic residues in the second pocket on Nup2 may be more important for this interaction.

### 2.7 Influence of Nup2 variants on the localization of importin‐α‐GFP

To assess the interaction between Nup2 and importin‐α in vivo, we took advantage of an integrated importin‐α‐GFP relocalization assay, which exploits the observation that when importin‐α‐GFP is expressed from an *SRP1‐GFP* gene integrated into the *SRP1* locus within the genome, the protein is primarily detected at the nuclear rim and not within the nuclear interior (Figure 7C). However, in cells that lack Nup2, this importin‐α‐GFP instead accumulates within the nuclear interior.^35^, ^36^, ^46^ If the amino acid changes in Nup2 impair the interaction with importin‐α in vivo, the integrated importin‐α‐GFP should be mislocalized to the nucleus in cells expressing the variant as the only copy of Nup2. Accordingly, integrated importin‐α‐GFP was localized in *ΔNUP2* cells containing either vector alone or plasmids encoding the Nup2 variants (Figure 7C). As expected, integrated importin‐α‐GFP is localized to the nucleus in cells lacking Nup2 and in cells expressing Δ50 Nup2, but is localized to the nuclear rim in cells expressing wild‐type Nup2. In cells expressing any of the Nup2 variants, integrated importin‐α‐GFP is mislocalized to the nucleus, suggesting that both of the importin‐α binding regions within Nup2 are important for a productive interaction in vivo.

### 2.8 Overexpression analysis

Reversing the charge of importin‐α Arg44 results in a loss of function (see Figure 3A) and further analysis suggests that this effect is due to impairment of the interaction of importin‐α Arg44 with Cse1 (see Figures 4A and 5), which could generate slower rates of cNLS‐cargo delivery in the nucleus. If this defect were indeed generated by a decline in the rate of cargo delivery, overexpression of a karyopherin release factor such as Nup2 or Cse1 could rescue the growth phenotype of the Arg44 importin‐α reversal‐of‐charge variants. Therefore, to test if overexpression of a karyopherin release factor can rescue the Arg44 importin‐α variant phenotypes, cells expressing either wild‐type importin‐α or importin‐α variants were transformed with plasmids that encode either Cse1 or Nup2 and were plated on control plates or plates containing 5‐FOA to remove the maintenance plasmid.^42^ An empty vector or a plasmid encoding importin‐β, which is not a known karyopherin release factor, was used as control.

**FIGURE 8:**
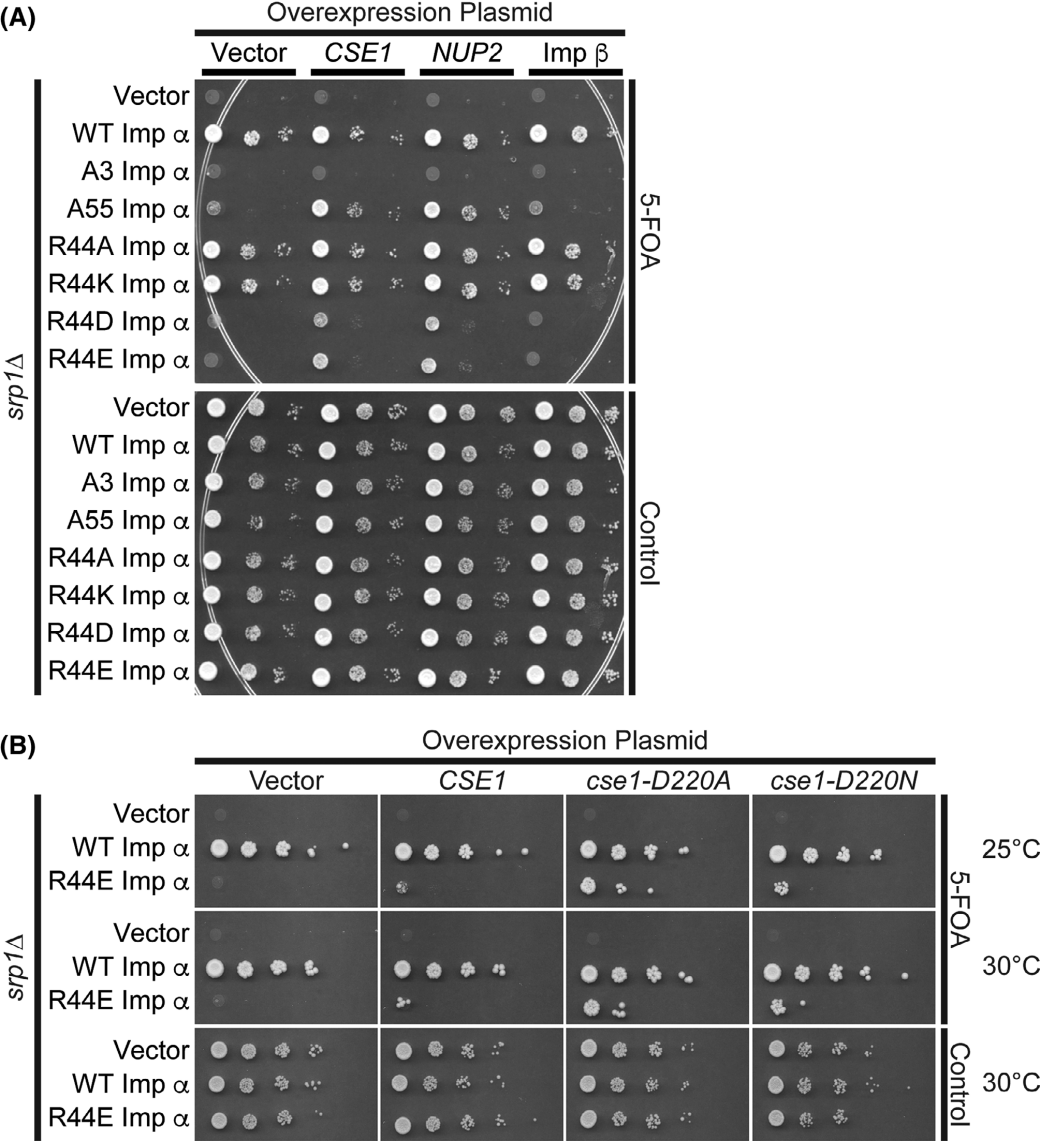
Overexpression analysis. A, *ΔSRP1* cells (ACY685) maintained by a plasmid encoding wild‐type importin‐α, expressing either wild‐type or variant importin‐α, and overexpressing Cse1, Nup2, or importin‐β were serially diluted, spotted onto control or 5‐FOA plates,^42^ and grown at the nonpermissive temperature. A vector control was also included. B, *ΔSRP1* cells (ACY685) maintained by a plasmid encoding wild‐type importin‐α, expressing either wild‐type or variant importin‐α, were transformed with plasmids overexpressing Cse1, Cse1‐D220A, or Cse1‐D220N. Cells were spotted and grown on control plates at 30°C, which maintains the *SRP1* maintenance plasmid, or 5‐FOA plates, to remove the maintenance plasmid, at 25°C and 30°C

As shown in Figure 8A, cells expressing wild‐type importin‐α grow well with and without overexpression of the KaRFs. Furthermore, as expected, cells expressing Arg44 variants that do not reverse the charge at this position, R44A or R44K importin‐α, display wild‐type growth. Although cells expressing the charge reversal variants, R44D or R44E importin‐α, as the sole copy of importin‐α are not viable, some modest growth is detected when either Cse1 or Nup2 is overexpressed in these cells. As controls, cells expressing A3 importin‐α, which has a major change in the autoinhibitory region and pronounced defects in cargo release,^29^ are not viable irrespective of which factors are overexpressed. Moreover, the cold‐sensitive phenotype of cells expressing A55 importin‐α, which has a smaller change in the autoinhibitory IBB region,^28^ is rescued by overexpression of either Cse1 or Nup2, but not importin‐β. This result supports the hypothesis that the Arg44 importin‐α substitution causes a defect in cNLS‐cargo release, because overexpression of not only Cse1, but also another karyopherin release factor, Nup2, can compensate to some extent for the effects of the reversal‐of‐charge and restore some function.

To extend this analysis, we tested whether variants of Cse1 that remove the charge at Asp220 in Cse1 could rescue growth of R44E importin‐α cells. Figure 8B shows that overexpression of either D220A or D220N Cse1 more effectively restores growth of R44E importin‐α cells as compared with wild‐type Cse1. This finding supports a model where these key residues are critical for the productive interaction between Cse1 and importin‐α, which results in efficient recycling of importin‐α to the cytoplasm following a round of nuclear import.

## 3 DISCUSSION

We have used complementary in vivo and in vitro methods to evaluate the contributions made by key residues in importin‐α, Cse1 and Nup2 to the interactions between these proteins and their function in nucleocytoplasmic transport. The crystal structures of Cse1/RanGTP/importin‐α and Nup2/importin‐α provided molecular models of how KaRFs might contribute to cNLS‐cargo release^40^, ^41^ that we have tested in vivo. In the trimeric export complex, Cse1 forms key interactions with both the C‐terminus and the IBB domain of importin‐α. In the present study, we have tested the hypothesis that the interaction between Arg44 of importin‐α and Asp220 of Cse1 is crucial for these two proteins to interact to form an export complex in vivo. We demonstrated that reversing the charge of either of these residues caused importin‐α or Cse1 to be nonfunctional and that the Arg44 importin‐α reversal‐of‐charge variants were unable to interact with Cse1 in vivo and in vitro (Figures 2 and 3). Similarly, change of Cse1 Asp220 to either Arg or Lys impaired its interaction with importin‐α and these Cse1 variants were also unable to interact productively with importin‐α in vivo. This loss of function is not due to a change in the level of these Cse1 variants (Figure 2D), suggesting that Asp220 is required in vivo for a functional interaction between Cse1 and importin‐α. Interestingly, D220N is the amino acid change present in the *cse1‐1* mutant. This mutant of *CSE1* was one of the first to be characterized and was found in a screen for chromosome segregation mutants,^13^ which gave the export factor its name. The cause of the *cse1‐1* phenotype had previously not been explored in detail, but our studies suggest that a defect in cNLS‐cargo delivery could contribute to the chromosome segregation errors, perhaps by impacting import of components critical for the segregation process.

The structural model of the N‐terminus of Nup2 bound to ΔIBB importin‐α showed that Nup2 interacts with two areas of importin‐α: the minor cNLS‐binding pocket and the C‐terminus.^40^, ^41^ We found that amino acid substitutions within Nup2 targeted to impact importin‐α binding to either or both of these regions caused a spectrum of growth defects and disrupted the interaction with importin‐α in vivo. These results are consistent with basic residues in the N‐terminus of Nup2 contributing to its function in vivo that was suggested by the crystal structure of the Nup2 importin‐α complex.^41^

Previously, two different models had been proposed to explain the role of Nup2 in cNLS‐cargo release. The first envisaged Nup2 functioning as a scaffold, facilitating cNLS‐cargo release and importin‐α recycling by enhancing the local concentrations of importin‐α, RanGTP and other dissociation factors at the NPC nuclear face.^41^ The second model envisaged Nup2 functioning analogously to the autoinhibitory domain, binding electrostatically to the C‐terminus of importin‐α, folding it over the groove of the minor cNLS‐binding pocket and extending toward the major cNLS‐binding pocket, thereby competing for binding with both mono‐ and bipartite cNLSs.^40^, ^41^ Nup2 itself could then be displaced from the C‐terminus of importin‐α by Cse1.^47^ The in vitro and in vivo results presented in this study support this second model, but do not preclude the first. For example, although the Nup2 N‐terminus competes for binding with the cNLS, the Nup2 Ran‐binding domain could recruit RanGTP to facilitate the dissociation of importin‐β and the freeing of the autoinhibitory domain, and the Nup2 FXFG repeats could recruit Cse1 to prepare for its role in dissociation and export.

The results presented here complement and extend single‐molecule fluorescence resonance energy transfer and particle tracking, which demonstrated that, in permeabilized HeLa cells, the complex between importin‐α and CAS (human Cse1) forms in the nuclear basket region of NPCs and appears to also involve the nuclear basket protein NUP50.^32^ Although the choreography of the individual proteins and complexes is quite complicated,^32^ results from the analysis performed in permeabilized HeLa cells also support the second model.

Taken together, structural, biochemical, and studies performed in cells and now in vivo provide insight into mechanisms that ensure efficient import of cargo proteins that contain a classical NLS into the nucleus. Future studies will focus on establishing whether cNLS‐cargo delivery in the nucleus occurs in a sequential or a concerted manner, advancing in a step‐wise progression or proceeding with collaborative, cooperative mechanism. This work also provides the groundwork for exploring the many additional nuclear transport pathways for both import and export that are mediated by other members of the importin‐β family of proteins. In many cases, the signals recognized by these receptors have not yet been defined so the detailed mechanistic understanding of the most well characterized pathway will provide a basis for future studies of these many routes by which cargoes enter and exit the nucleus, including understanding how these pathways are regulated. Such studies are critically important as both nuclear import and export pathways are emerging as promising pathways to target in chemotherapy.^48^ Exploiting these pathways for therapeutic purposes will require a detailed mechanistic understanding of the various interactions that are critical at each step.

## 4 MATERIALS AND METHODS

### 4.1 Strains, plasmids and chemicals

All chemicals were obtained from US Biological or Sigma unless noted otherwise. All media were prepared and all DNA manipulations were performed according to standard procedures.^49^ All *S*. *cerevisiae* strains and plasmids used in this study are summarized in Table 1. The *SRP1* gene encodes importin‐α, *CSE1* encodes Cse1, and *NUP2* encodes Nup2.

**TABLE 1:** Strains and plasmids used in this study

Strain/plasmid	Description	Origin/reference
FY23 (ACY192)	Wild‐type, *MATa ura3‐52 leu2Δ1 trp1*	51
ACY324	*ΔSRP1::HIS3 [SRP1 CEN URA3 Amp^*R*^*], *MATα leu2 lys*	29
ACY685	*ΔSRP1::HIS3 [SRP1 CEN URA3 Amp* ^*R*^], *MATa leu2 trp1*	This study
ACY712	*ΔNUP2*::*KAN importin α‐GFP::HIS3* [*SRP1 CEN URA3 Amp* ^*R*^], *leu2*	41
ACY789	*ΔNUP2 srp1‐31* [*SRP1 CEN URA3 Amp* ^*R*^], *MATa ura3 leu2 his3 trp1 lys*	41
ACY1237	*ΔCSE1*::*KAN* [*CSE1 CEN URA3 Amp* ^*R*^], *MATa his3Δ1 leu2Δ0*	This study
ACY1552	*ΔNUP2::HIS3ΔNUP133*::*KAN* [*NUP2 CEN URA3 Amp* ^*R*^], *MATa leu2 ura3*	This study
pRS315 (pAC3)	*CEN LEU2 Amp* ^*R*^	52
pRS305 (pAC7)	*2μ LEU2 Amp* ^*R*^	52
pAC592	Importin β, *2μ TRP1 Amp* ^*R*^	29
pAC855	Importin α A3, *CEN LEU2 Amp* ^*R*^	29
pAC856	Importin α, *CEN LEU2 Amp* ^*R*^	29
pAC858	Importin α A55, *CEN LEU2 Amp* ^*R*^	29
pAC883	Importin α‐GFP, *CEN URA3 Amp* ^*R*^	29
pAC890	Importin α‐GFP A3, *CEN URA3 Amp* ^*R*^	29
pAC891	Importin α‐myc, *CEN URA3 Amp* ^*R*^	29
pPS1176 (pAC958)	Cse1, *2μ LEU2 Amp* ^*R*^	P.A. Silver
pAC1215	1–51 Nup2‐GFP, *CEN LEU2 Amp* ^*R*^	41
pAC1268	Nup2 Δ50, *CEN LEU2 Amp* ^*R*^	41
pAC1271	Cse1‐GST, *Amp* ^*R*^	P.A. Silver
pAC1298	Nup2, *CEN URA3 Amp* ^*R*^	41
pAC1303	Cse1, *2μ TRP1 Amp* ^*R*^	29
pAC1342	Nup2, *CEN LEU2 Amp* ^*R*^	41
pAC1385	Nup2, *2μ TRP1 Amp* ^*R*^	29
pAC1394	Nup2‐GFP Δ50, *2μ URA3 Amp* ^*R*^	41
pAC1395	Nup2‐GFP, *2μ URA3 Amp* ^*R*^	This study
pAC1880	Importin α R44A, *CEN LEU2 Amp* ^*R*^	This study
pAC1881	Importin α R44E, *CEN LEU2 Amp* ^*R*^	This study
pAC1882	Importin α R44D, *CEN LEU2 Amp* ^*R*^	This study
pAC1883	Importin α R44K, *CEN LEU2 Amp* ^*R*^	This study
pAC1884	Importin α‐GFP R44A, *CEN URA3 Amp* ^*R*^	This study
pAC1885	Importin α‐GFP R44E, *CEN URA3 Amp* ^*R*^	This study
pAC1886	Importin α‐GFP R44D, *CEN URA3 Amp* ^*R*^	This study
pAC1887	Importin α‐GFP R44K, *CEN URA3 Amp* ^*R*^	This study
pAC1892	Cse1 D220A, *2μ LEU2 Amp* ^*R*^	This study
pAC1893	Cse1 D220R, *2μ LEU2 Amp* ^*R*^	This study
pAC1894	Cse1 D220K, *2μ LEU2 Amp* ^*R*^	This study
pAC1895	Cse1 D220N, *2μ LEU2 Amp* ^*R*^	This study
pAC1896	Cse1‐GST D220A, *Amp* ^*R*^	This study
pAC1897	Cse1‐GST D220R, *Amp* ^*R*^	This study
pAC1898	Cse1‐GST D220K, *Amp* ^*R*^	This study
pAC1899	Cse1‐GST D220N, *Amp* ^*R*^	This study
pAC3536	Cse1‐GFP, *2μ TRP1 Amp* ^*R*^	This study
pAC3537	Cse1‐GFP D220A, *2μ TRP1 Amp* ^*R*^	This study
pAC3538	Cse1‐GFP D220R, *2μ TRP1 Amp* ^*R*^	This study
pAC3539	Cse1‐GFP D220K, *2μ TRP1 Amp* ^*R*^	This study
pAC3540	Cse1‐GFP D220N, *2μ TRP1 Amp* ^*R*^	This study
pAC2283	Nup2 K3E/R4D, *CEN LEU2 Amp* ^*R*^	This study
pAC2286	Nup2 R38A/R39A R46A/R47A, *CEN LEU2 Amp* ^*R*^	This study
pAC2289	Nup2 K3E/R4D R38A/R39A R46A/R47A, *CEN LEU2 Amp* ^*R*^	This study
pAC2290	Nup2‐GFP K3E/R4D, *2μ URA3 Amp* ^*R*^	This study
pAC2293	Nup2‐GFP R38A/R39A R46A/R47A, *2μ URA3 Amp* ^*R*^	This study
pAC2296	Nup2‐GFP K3E/R4D R38A/R39A R46A/R47A, *2μURA3 Amp* ^*R*^	This study
pAC1900	Cse1 D220A, *2μ TRP1 Amp^R^*	This study
pAC1903	Cse1 D220N, *2μ TRP1 Amp^R^*	This study

### 4.2 Microscopy

GFP‐fusion proteins were localized in live cells using direct fluorescence microscopy to visualize the fluorescently‐tagged proteins in living cells. The GFP signal was visualized using a GFP‐optimized filter (Chroma Technology) on an Olympus BX60 epifluorescence microscope equipped with a Photometrics Quantix digital camera. Cells expressing GFP‐tagged proteins from their own promoters were grown overnight in selective media, diluted in fresh media, and grown for 3 hours prior to localization studies.

### 4.3 Immunoblotting

Protein samples (30‐50 μg lysate; total bound) were resolved on Criterion 4% ‐20% gradient gels (Bio‐Rad), transferred to nitrocellulose membranes (Bio‐Rad) and Myc‐tagged proteins were detected with anti‐Myc monoclonal antibody 9B11 (1:2000; Cell Signaling), GFP‐tagged proteins were detected with anti‐GFP rabbit polyclonal antibody (1:3000; Sigma), and GST fusion proteins were detected with anti‐GST monoclonal antibody (1:2000; Santa Cruz Biotechnology).

### 4.4 In vivo functional analysis

The in vivo function of each of the importin‐α, Nup2, or Cse1 variants was tested using a plasmid shuffle technique.^42^ Plasmids encoding wild‐type or mutant importin‐α, Nup2 or Cse1 were transformed into *ΔSRP1* (ACY324), *ΔCSE1* (ACY1237) or *ΔNUP2/ΔNUP133* (ACY1552) cells containing a wild‐type *SRP1*, *CSE1*, or *NUP2 URA3* plasmid. Single transformants were grown to saturation in liquid culture, serially diluted (1:10) in dH_2_O, and spotted on control ura‐ leu‐ glucose plates or on selective leu‐ glucose plates containing 5‐fluoroorotic acid (5‐FOA) (ThermoFisher Scientific, catalog # R0811). Plates were incubated at 18°C, 25°C, 30°C or 37°C as indicated for 3 to 5 days. To clarify the results of the Nup2 functional assay, cells were picked from the 25°C 5‐FOA plate, grown to saturation at 25°C, serially diluted, spotted on glucose plates lacking leucine and incubated at 30°C.

### 4.5 In vitro binding assays

For the Cse1/importin‐α binding assay, Cse1‐GST beads were incubated with yeast lysates expressing importin‐α‐myc variants. To prepare Cse1‐GST beads, *Escherichia coli* cells expressing wild‐type Cse1‐GST (pAC1271) were resuspended in PBS supplemented with 0.5 mM phenylmethylsulfonyl fluoride, PLAC (3 mg/mL each of pepstatin A, leupeptin, aprotinin, chymostatin), and 2 mM β‐mercaptoethanol and lysed by sonication. Lysates were cleared by centrifugation and incubated with 200 μL of prepared glutathione‐Sepharose beads (GE Healthcare/MillaporeSigma, catalog # GE17‐0756‐01). Beads were collected and washed three times with PBS then incubated with yeast lysate. To prepare yeast lysates that contain myc‐tagged variants of importin‐α, wild‐type cells (ACY192) containing plasmids encoding wild‐type or variant importin‐α‐myc (pAC894, pAC891, pAC1888, pAC1889, pAC1890, pAC1891) were collected and washed twice in dH_2_O and once in PBSMT (PBS, 5 mM MgCl_2_, 0.5% Triton X‐100). Glass bead lysis was conducted in PBSMT supplemented with protease inhibitors (phenylmethylsulfonyl fluoride and PLAC). Lysates were cleared by centrifugation and total protein concentration was assessed by Bradford assay. Two milligrams of total yeast lysate and 50 μL of Cse1‐GST bead slurry were incubated overnight with agitation. The unbound fraction was removed and the bound fraction was washed twice with PBSMT and twice with PBS. Bound and unbound samples were loaded on a 10% denaturing SDS‐PAGE gel. The proteins were transferred to a nitrocellulose membrane and probed with either primary α‐GST (mouse, Santa Cruz Biotechnology B‐14, catalog #sc‐138) or primary α‐myc (mouse, Cell Signaling 9B11, catalog #2276) and secondary α‐mouse HRP‐conjugated antibodies to detect the Cse1‐GST or importin‐α‐myc proteins.

For the Nup2/importin‐α binding assay, c‐myc beads were incubated with lysates from cells expressing both Nup2‐GFP and importin‐α‐myc. Lysates from wild‐type cells (ACY192) containing plasmids encoding wild‐type or variant Nup2‐GFP (pAC1394, pAC1395, pAC2290, pAC2293, pAC2296) and wild‐type importin‐α‐myc (pAC2326) were prepared as described above. One milligram of yeast lysate was incubated overnight with 20 μL prepared c‐myc beads (Santa Cruz Biotechnology) in a total volume of 500 μL PBSMT. Fractions were prepared as above, loaded on a 10% denaturing SDS‐PAGE gel, transferred to a nitrocellulose membrane, and probed with either primary α‐GFP (rabbit) and secondary α‐rabbit HRP‐conjugated antibodies or primary α‐myc (mouse, Cell Signaling) and secondary α‐mouse HRP‐conjugated antibodies to detect the Nup2‐GFP or importin‐α‐myc proteins.

### 4.6 High copy suppression growth assays

To assess suppression of the loss of viability of cells expressing nonfunctional importin‐α variants as the sole copy of importin‐α, cells containing importin‐α variants as *LEU2* plasmids as well as a *URA3* importin‐α maintenance plasmid were transformed with the following high copy 2μ *TRP1* plasmids encoding; vector (control), Cse1, Cse1 variants (Cse1‐D220A, Cse1‐D220N), Nup2, or importin‐β and selected on Ura‐Leu‐Trp‐ minimal media. Cells were grown overnight at 25°C to saturation in Leu‐Trp‐ minimal media, cell concentrations were normalized by OD_600_, and cells were serially diluted and spotted onto control Ura‐Leu‐Trp‐ minimal media (to maintain the wild‐type copy of importin‐α) or Leu‐Trp— minimal media containing 5‐FOA. Growth was examined at 25, 30 and 37°C. Data are presented for the nonpermissive temperature.

## CONFLICT OF INTEREST

The authors declare no conflict of interest.
